# Assessment of Burnout and its Factors Among Doctors Using the Abbreviated Maslach Burnout Inventory

**DOI:** 10.7759/cureus.4101

**Published:** 2019-02-19

**Authors:** Altaf A Shaikh, Anam Shaikh, Rajesh Kumar, Amber Tahir

**Affiliations:** 1 Internal Medicine, Ghulam Mohammad Mahar Medical College and Hospital, Sukkur, PAK; 2 Family Medicine, Ghulam Mohammad Mahar Medical College and Hospital, Sukkur, PAK; 3 Internal Medicine, Dow University of Health Sciences, Karachi, PAK

**Keywords:** maslach burnout inventory, physician burnout, emotional exhaustion, depersonalization, personal accomplishment, job dissatisfaction, abbreviated maslach burnout inventory, burnout dimension, burnout syndrome

## Abstract

Background

Occupational burnout is an unwanted outcome of chronic workplace stressors which may be emotional or interpersonal. Chronic exposure to human suffering and long working hours have contributed to greater job stress and early burnout among healthcare providers. This study utilized the abbreviated Maslach Burnout Inventory (aMBI) to gauge the extent of overall burnout and on three subscales – perspective taking, compassionate care, and walking in patients' shoes – among interns, postgraduate trainees, and physicians of internal medicine.

Materials and methods

In this cross-sectional study, 71 internal medicine doctors – 40 interns, 22 postgraduate trainees, and nine physicians – completed aMBI with informed consent. It is a nine-item scale with three subscales – emotional exhaustion (EE), depersonalization (DP), and personal accomplishment (PA). Each subscale has three items that are marked on a seven-point Likert scale. Higher scores of EE and DP indicate higher burnout, and a higher score of PA indicates lower burnout. Overall burnout was taken as the sum of EE and DP. Data were entered and analyzed using SPSS v. 22.

Results

There were 23 (32.4%) male and 48 (67.5%) female doctors with a mean age of 24.25 ± 13.17 years. The mean score of overall burnout was 22.51 ± 6.07 (range: 0–36) and PA was 15.35 ± 1.82 (range: 0–18). Overall moderate to high burnout was seen in 33.8% of doctors. On an individual subscale, 47.8% had high EE, 24% had high DP, and 25.4% reported high burnout on PA. Overall burnout had a statistically significant correlation with the marital status of the doctors, their working hours per week, their average on-call days per week, and their level of expertise.

Conclusion

There is a high degree of burnout among internal medicine physicians. Working hours and the number of on-call days per week were significant predicting factors. Interns reported the highest frequency of burnout.

## Introduction

The phenomenon of occupational burnout was first studied in 1970s. It has taken under its umbrella a wide range of emotions including exasperation, irritation, anger, and hopelessness which results in negative and dismissive attitude towards the occupation and people related to it. Burnout has been more commonly seen (or studied) in occupations that demand constant human to human interaction [1]. Occupational burnout is an unwanted outcome of chronic workplace stressors which may be emotional or interpersonal [2]. Burnout has been studied among practitioners of healthcare at all levels including medical students, nurses, interns, postgraduate trainees, and special care providers [2-5].

Although healthcare is a noble and rewarding profession, but chronic exposure to human suffering and long working hours have contributed to greater job stress and early burnout among the healthcare providers [1].

Maslach and Jackson have described burnout as a state of emotional exhaustion, depersonalization, and reduced sense of accomplishment in people who do some sort of people-related work [6]. Hence, burnout is a multidimensional syndrome that deprives people off of their emotional health and well-being. Burnout leads to a cynical approach towards concerned people and decreases the perception of personal accomplishments [7].

A recent elaborated systemic review of 182 studies published in the Journal of the American Medical Association (JAMA) reported an overall burnout prevalence of 67% among physicians. Emotional exhaustion was seen in 72% studies, depersonalization was reported in 68.1% studies, and compromised personal accomplishment was shown in 63.2% studies [8].

This study utilized the abbreviated version of Maslach Burnout Inventory (aMBI) to gauge the extent of burnout on the three above-mentioned subscales among interns, postgraduate trainees, and physicians of internal medicine.

## Materials and methods

This cross-sectional study was conducted in the Department of Internal Medicine, Ghulam Mohammad Mahar Teaching Hospital, Pakistan in December 2018. The sampling technique used was convenient sampling. There were 71 doctors in the department at that time. All of them completed the study. Informed consent was obtained from all the doctors and they were requested to complete the questionnaire given to them. Upon attaining consent, the aims of this study were briefly explained to them. All information provided by the doctors was kept confidential and was only utilized for the purpose of this study.

The questionnaire comprised two sections. The first section included demographic characteristics of the doctors including gender, age, living status (away from the family for work or with the family), marital status, and the number of working hours per week. On average, the interns and postgraduate trainees work 80 hours per week and the physicians work 48 hours per week. Section two of the questionnaire comprised the aMBI. aMBI is a nine-item scale used for assessing burnout. It has three subscales – emotional exhaustion, (EE, emotional depletion due to job demand and continuous work-related stress), depersonalization (DP, impersonal response toward the recipient service), and personal accomplishment (PA, the degree of personal competence, achievement, and satisfaction with work). Each subscale is assessed by three items. For each item, there is a seven-point Likert scale which ranges from never (0) to every day (6). The score for each item was summed up for each doctor. For emotional exhaustion and depersonalization, higher score means greater burnout; this is inverse for personal accomplishment. The score of each subscale could range from minimum 0 to maximum 18. High score of EE and DP and a lower score of PA indicates a higher level of burnout.

Data analysis was performed using statistical package for Social Sciences (SPSS) version 22. The validity and reliability of aMBI have already been established [9]. Internal reliability of each subscale of aMBI was calculated using Cronbach’s alpha coefficient. For emotional exhaustion α = 0.89, for depersonalization α= 0.76, for personal accomplishment α = 0.72, and for overall burnout α = 0.81. Overall burnout was taken as the sum of scores of EE and DP [10]. For EE and DP, subscale score of 0–9 was categorized as “no to low burnout” and subscale score of 10–18 was regarded as “moderate to severe burnout.” It was the opposite for PA because higher PA scores indicate lesser burnout. Frequencies and percentages were calculated for demographic variables. Mean and standard deviation (SD) were calculated for continuous variables including age and working hours. Differences in the frequency of burnout were deduced using the chi-squared test. A p-value <0.05 was used as the level of significance.

## Results

Out of the 71 doctors who completed the study, there were 40 interns, 22 postgraduate residents, and nine consultant physicians. Their mean age was 24.25 ± 13.17 years. The distribution and frequency of their gender, ages, marital status, on-call days/week, and working hours/week are shown in Table 1.

**Table 1:** Frequency of demographic and work-related characteristics of the doctors (N = 71)

PARTICIPANT CHARACTERISTICS	FREQUENCY (%)
GENDER	
Male	23 (32.4%)
Female	48 (67.5%)
MARITAL STATUS	
Single	54 (76%)
Married	15 (21.1%)
Divorced	2 (2.8%)
Widow	--
AGE (in years)	
≤25 years	50 (70.5%)
25-35 years	17 (23.9%)
≥35 years	4 (5.6%)
WORKING HOURS PER WEEK	
≤60 hours	16 (22.5%)
≥60 hours	55 (77.5%)
AVERAGE ON-CALL DAYS PER WEEK	
≤2 days	56 (78.8%)
>2 days	15 (21.2%)

The scores of aMBI were computed for each subscale and combined for EE and DP. Higher scores indicate higher burnout except for PA, where higher scores indicate lesser burnout. The mean ± SD of each subscale score and overall burnout and categorized as “not to low” and “moderate to high” are shown in Table 2. Overall moderate to high burnout was seen in 33.8% doctors, 47.8% had high emotional exhaustion, and 24% had high depersonalization. There were 74.6% doctors who were satisfied with their personal accomplishments, and 25.4% reported moderate to high burnout on PA (Table 2).

**Table 2:** Abbreviated Maslach Burnout Inventory Scores and their percentiles

aMBI SUBSCALE	SCORE (MEAN ± SD)	NO TO LOW BURNOUT N (%)	MODERATE TO HIGH BURNOUT N (%)
Emotional exhaustion (EE)	12.69 ± 3.05	37 (52.2%)	34 (47.8%)
Depersonalization (DP)	9.87 ± 4.28	54 (76.0%)	17 (24.0%)
Personal accomplishment (PA)	15.35 ± 1.82	53 (74.6%)	18 (25.4%)
Emotional exhaustion + Depersonalization = overall burnout (OB)	22.51 ± 6.07	47 (66.2%)	24 (33.8%)

Nineteen interns (47.5%) reported moderate to high burnout, four (18.2%) postgraduate trainees moderate to high burnout, and one (11.1%) consultant physician reported moderate to high burnout. Overall burnout among the doctors was stratified according to their demographic and work-related characteristics, and it was seen that overall burnout had a statistically significant correlation with the marital status of the doctors, their working hours per week, their average on-call days per week, and their level of expertise as shown in Table 3.

**Table 3:** Relationship of demographic and work-related characteristics of the doctors with overall burnout

DOCTOR’S CHARACTERISTICS	OVERALL BURNOUT				P-VALUE (<0.05 SIGNIFICANT)
	NO TO LOW BURNOUT n (%)			MODERATE TO HIGH BURNOUT n (%)	
GENDER					0.13
Male		18 (38.3%)		5 (20.8%)	
Female		29 (61.7%)		19 (79.2%)	
MARITAL STATUS					0.04
Single		40 (85.1%)	14 (58.3%)		
Married		6 (12.7%)	9 (37.5%)		
Divorced		1 (2.1%)	1 (4.1%)		
Widow		--	--		
AGE (in years)					0.40
≤25 years		35 (74.4%)	15 (62.5%)		
25-35 years		9 (19.1%)	8 (33.3%)		
≥35 years		3 (6.4%)	1 (4.2%)		
WORKING HOURS PER WEEK					0.04
≤60 hours		14 (29.7%)	2 (8.3%)		
≥60 hours		33 (70.2%)	22 (91.6%)		
AVERAGE ON-CALL DAYS / WEEK					<0.0000
≤2 days		44 (93.6%)	12 (50%)		
>2 days		3 (6.4%)	12 (50%)		
LEVEL OF EXPERTISE					0.04
Interns		21 (44.6%)	19 (79.1%)		
Postgraduates		18 (38.3%)	4 (16.6%)		
Consultant physicians		8 (17%)	1 (4.1%)		

## Discussion

Healthcare is an emotionally challenging job that demands empathy. Healthcare professionals (HCPs) are vulnerable to emotional depletion and sense of detachment from the profession. Burnout percentages were higher in interns than postgraduate trainees and consultant physicians. The outcomes of this study highlight that more than one-quarter of HCPs have moderate to high burnout. Longer working hours and more on-call days per week were significant contributing factors. Interns and postgraduates were more burnt out than consultant physicians. More than a shocking finding, burnout is an inevitable side effect of a profession that has always glamorized the myth of invulnerability and the survival of the fittest. This educational system has propagated rewards of self-denial and expert performance in face of gigantic work performance pressure.

Shanafelt T et al., in a national survey, reported almost half of the American physicians to be experiencing some degree of burn out. HCPs of emergency medicine, internal medicine, and family medicine were reported to be at highest risk [11]. Without addressing burnout, physicians experience a downward spiral in their personal and professional lives. It doesn’t only present with job dissatisfaction but also leads to relationship issues, substance abuse, and even suicidal ideation. It also has detrimental effects on patient care and safety which is a leading concern. Physician burnout causes distracted behavior, chances of medical errors, and malpractice risks, which not only decreases patient satisfaction but also compromises the quality of care and disease outcome [12]. It has been established that there are lesser complications in patients whose physicians are more empathic [13].

In Japan, 225 physicians have been reported to be emotionally exhausted, 11% with high depersonalization and 62% with decreased personal accomplishment [14]. Studies with family physicians in Europe have shown 43% EE, 35% DP, and 32% reduced PA [15-16]. The overall burnout among Saudi physicians has been reported to be 25.2%, with 69.5% physicians emotionally exhausted, 26% depersonalized, and 12.2% of physicians felt the lack of personal accomplishment [1]. In internal medicine and pulmonology trainees of Mirpur, early to advanced burnout was reported in 26.5% postgraduate trainees [2]. The extrinsic occupational factors predicting overall burnout and job satisfaction among Turkish doctors included vacation days at the personal level and institutional ownership at the group level. The work-related factor predicting burnout was the number of shifts per month [3].

The outcomes of this study and those done before this converge to one common need – the need for assistance for the one responsible for the provision of healing and care. Despite the stigma attached to seeking psychosocial help, it has been seen that successful multidimensional interventions targeting range of burnout triggers and incorporating a variety of tools will help reduce burnout rate among healthcare providers. Many interventional studies have been conducted which have reported that restricting the number of working hours of the trainees resulted in increased career satisfaction and decreased burnout on the subscale of emotional exhaustion; however, the concerns with patient care quality and doctors; learning and education also increased with reduced work hours [17-18]. Besides, strategies such as stress-management workshops have also been helpful. In an interventional study with pediatric and Med-Ped residents, a four-hour long workshop was conducted which targeted four aspects of emotional intelligence (EI) among the residents – self-awareness, self-management, social awareness, and social skills. The residents completed an online EI-survey before and after the intervention. A significant increase in median total EI score, composite median stress management score, and overall wellness score was observed [19]. Such interventions targeting behavioral and cognitive principles are our most promising evidence-based approach to reduce stress and burnout among doctors.

## Conclusions

There is a high degree of burnout among internal medicine physicians. Working hours and number of on-call days per week were significant correlating factors. Interns reported the highest frequency of burnout. Multidimensional interventions that can target a wide range of burnout triggers by incorporating various therapeutic tools can help in reducing burnout rate among healthcare providers.
